# Modeling and control of a bedside cable-driven lower-limb rehabilitation robot for bedridden individuals

**DOI:** 10.3389/fbioe.2023.1321905

**Published:** 2023-11-23

**Authors:** Daoyu Wang, Jicai Li, Zhuo Jian, Hao Su, Hongbo Wang, Fanfu Fang

**Affiliations:** ^1^ Academy for Engineering and Technology, Fudan University, Shanghai, China; ^2^ Shanghai ZD Medical Technology Co., Ltd., Shanghai, China; ^3^ Department of Mechanical and Aerospace Engineering, North Carolina State University, Raleigh, NC, United States; ^4^ Joint NCSU/UNC Department of Biomedical Engineering, North Carolina State University, Raleigh, NC, United States; ^5^ University of North Carolina at Chapel Hill, Chapel Hill, NC, United States; ^6^ Department of Rehabilitation Medicine, Changhai Hospital, Shanghai, China

**Keywords:** cable-driven, lower-limb rehabilitation, human–machine coupling, impedance identification, sling exercise therapy

## Abstract

Individuals with acute neurological or limb-related disorders may be temporarily bedridden and unable to go to the physical therapy departments. The rehabilitation training of these patients in the ward can only be performed manually by therapists because the space in inpatient wards is limited. This paper proposes a bedside cable-driven lower-limb rehabilitation robot based on the sling exercise therapy theory. The robot can actively drive the hip and knee motions at the bedside using flexible cables linking the knee and ankle joints. A human–cable coupling controller was designed to improve the stability of the human–machine coupling system. The controller dynamically adjusts the impedance coefficient of the cable driving force based on the impedance identification of the human lower-limb joints, thus realizing the stable motion of the human body. The experiments with five participants showed that the cable-driven rehabilitation robot effectively improved the maximum flexion of the hip and knee joints, reaching 85° and 90°, respectively. The mean annulus width of the knee joint trajectory was reduced by 63.84%, and the mean oscillation of the ankle joint was decreased by 56.47%, which demonstrated that human joint impedance identification for cable-driven control can effectively stabilize the motion of the human–cable coupling system.

## 1 Introduction

Flaccidity after a stroke is the first stage in the Brunnstrom stages of stroke recovery, also known as flaccid paralysis (Cruz-Almeida et al., 2005; Baer et al., 2014). In flaccid paralysis after cerebral hemorrhage for 2–3 weeks, the patients are conscious or have mildly impaired consciousness, and the vital signs are stable (Dunkerley et al., 2000; Hendricks et al., 2002; Kvorning et al., 2006). However, the muscle strength and tone of the affected limbs and the tendon reflexes are low (Meythaler et al., 2001; Cao et al., 2023). Rehabilitation nursing measures should be undertaken early so as not to interfere with clinical resuscitation and not cause deterioration of the condition. The objective is to prevent complications and secondary injury while preparing for the next step of functional rehabilitation training (Singer and Mochizuki, 2015; Yang et al., 2023). Sling training effectively improves joint movement and reduces muscle tissue damage in the post-stroke period of flaccid paralysis (Lee and Lee, 2014). Sling exercise therapy (SET) is an unstable chain movement performed with a suspension aid to improve the stability of the core muscles (Oh and Kwon, 2017). Some rehabilitation treatments use the SET to enhance the proprioception, balance, neuromuscular control, and walking ability of individuals with flaccidity (Coote et al., 2008; Jung and Choi, 2019). There are two kinds of kinetic chain exercises: open-chain and closed-chain (Jung and Choi, 2019). In open-chain training, the segment furthest from the body is free and not fixed to an object. In closed-chain training, the segment furthest away from the body is fixed. SET emphasizes the active participation of the patient in training and has both diagnostic and therapeutic functions (Charles et al., 2006). It is useful for detecting the weakest muscles in the human kinetic chain and strengthening them by performing closed-chain and open-chain exercises.

Previously, sling therapists assisted inpatients in moving the upper and lower extremities using elastic cables in a suspension frame (Park and Hwangbo, 2014). A clinical experiment with 50 stroke inpatients showed that upper limb motor dysfunction and shoulder pain are more effectively relieved with SET than with routine training within 2 months after stroke (Liu et al., 2020). Oh and Kwon (2017) applied the methods of sling exercises under the provision of vibrations for people with myelopathy and verified the effectiveness of a muscle function improvement program by sling exercise training (Oh and Kwon, 2017). Traditional sling exercise training has two major deficiencies, lack of feedback on human–machine interaction and non-intelligent training data feedback. Lower-limb rehabilitation sling training mainly depended on the assistance of therapists due to the loss of joint control function in patients in the early stage of stroke (Park and Hwangbo, 2014). Moving such patients from wards to treatment zones added to the trainers’ workload (Burtin et al., 2009). A bedside lower-limb rehabilitation training robot can reduce the work intensity of the therapists if it can easily move in the limited space of the ward (Barrett et al., 2006; Jackson et al., 1998). Several industrial and medical robotic manufacturers developed rehabilitation robots for bedridden patients, although there is no SET implemented by bedside rehabilitation robots for flaccid patients. Focusing on mobilizing bedridden patients to a new level, KUKA helps bedridden patients with early and efficient mobilization and relieves the healthcare professionals from heavy lifting and inconvenient working postures with a seven-axis manipulator (LBR Med) (KUKA, 2008). Yaskawa Electric Corporation proposed a bedside therapeutic device for the lower extremity therapy in cerebrovascular patients, which made it possible to repeat lower-limb joint training at varying speed settings and range of motion (Tsuda, 2006). Bedside rehabilitation robots are characterized by three main features: compact size, ease of mobility, and clear functionality. During passive rehabilitation training, the two bedside robots perform with high position accuracy in the workspace. However, both rely on a rigid human–machine coupling, with safety risks, while SET is based on a flexible human–cable coupling system.

Sling exercise training is an interactive process between the cables and the patient’s extremities. Research on substituting therapists with robots in sling exercise training is a helpful direction. Cable-driven robots are mechanisms in which the end-effector is moved by controlling the length of the cables connected to it (Zanotto et al., 2014; Li et al., 2021; Vashista et al., 2016; Xie et al., 2021). Cable-driven robots are appealing due to their structural simplicity, high torque-to-weight ratio, and flexibility (Liu et al., 2022; Rosati et al., 2007; Zarebidoki et al., 2021). Sophia-3 is a planar cable-driven device with a tilting working plane featuring a moving pulley block that allows the robot to achieve excellent force capabilities, despite the low number of cables (Zanotto et al., 2014). Its implemented force field could significantly improve users’ performance in terms of movement accuracy and execution time. NeReBot is a device used for the treatment of post-stroke upper-limb impairments based on cable transmission and direct-drive actuation (Masiero and Boschetti, 2017; Cao et al., 2022). These cable-driven rehabilitation robots can provide many benefits compared with devices characterized by a rigid structure, such as lower costs, reduced complexity, compliance by design, and a higher degree of reliability and safety. They can effectively move the patient’s upper and lower extremities within the training space (Lambert et al., 2020; Li and Zanotto, 2019). However, these robots take up a lot of space, which is unsuitable for application in patient wards. In addition, cable-driven robots are continuously unstable. Currently, there is no research on open- and closed-chain techniques to improve patients’ joint motion stability and neuromuscular control.

In this paper, we proposed a cable-driven lower-limb rehabilitation robot (SmartSling) for sling exercise therapy, especially aimed at inpatients in the phase of flaccid paralysis after stroke. As described previously, the contribution seems to be the design, and then it is described as the modeling and control. The contribution of this work is twofold: first, we modeled closed-chain kinematics and kinetics for the human–machine coupled system, and second, we proposed a human–cable impedance controller to minimize the hysteresis of knee movement and stabilize the interaction force for active sling training.

## 2 Modeling and control of SmartSling

### 2.1 Mechatronic system of SmartSling

**FIGURE 1:**
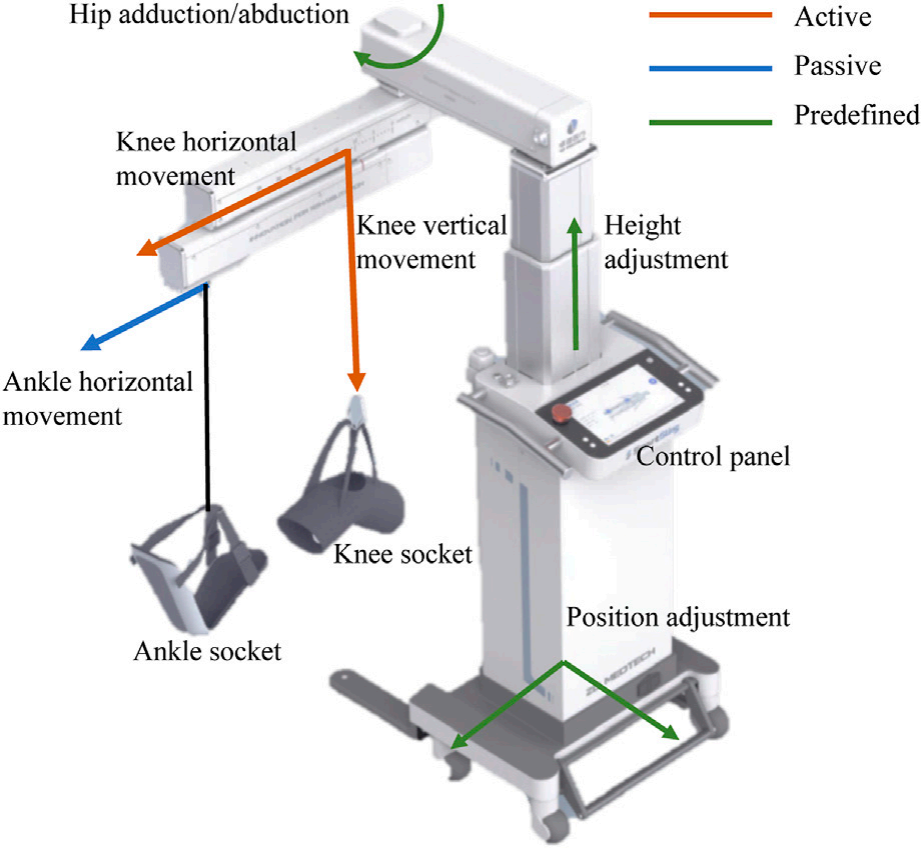
Caption system overview of the bedside cable-driven lower-limb rehabilitation robot, SmartSling. It consists of nine parts: knee horizontal movement module, knee vertical movement module, ankle horizontal movement module, knee socket, ankle socket, hip abduction and abduction module, height adjustment module, position adjustment module, and control panel. The knee vertical and horizontal movements are active (red), and the ankle horizontal movement is passive (passive). In addition, with the predefined modules, i.e., hip position and height adjustment modules (green), the rehabilitation robot can assist patients with bedside rehabilitation in most space-limited wards.

The design of the SmartSling lower-limb rehabilitation training system is shown in Figure 1. SmartSling comprises five components: a height adjustment module, a module for hip adduction–abduction, a module for horizontal–vertical movement in the sagittal plane with suspension cables, a knee–ankle socket, and a control panel for therapists. The modules for hip adduction–abduction and for horizontal–vertical movement are actuated by two DC servo motors (PD4-CB59M024035-E, Nanotec Electronic GmbH & Co. KG) via ball screw mechanisms. The cable connected to the knee joint has two active degrees of freedom in the sagittal plane. The cable connected to the ankle joint has only one passive degree of freedom, and its robotic side is constrained in a linear chute. The two cables are connected with six-axis force/torque sensors (M3715A, Sunrise Instruments). SmartSling can train the bedside inpatients in most space-limited wards, and its position can be adjusted thanks to a wheeled chassis and the height adjustment module.

### 2.2 Equilibrium state of basic kinematics

**FIGURE 2:**
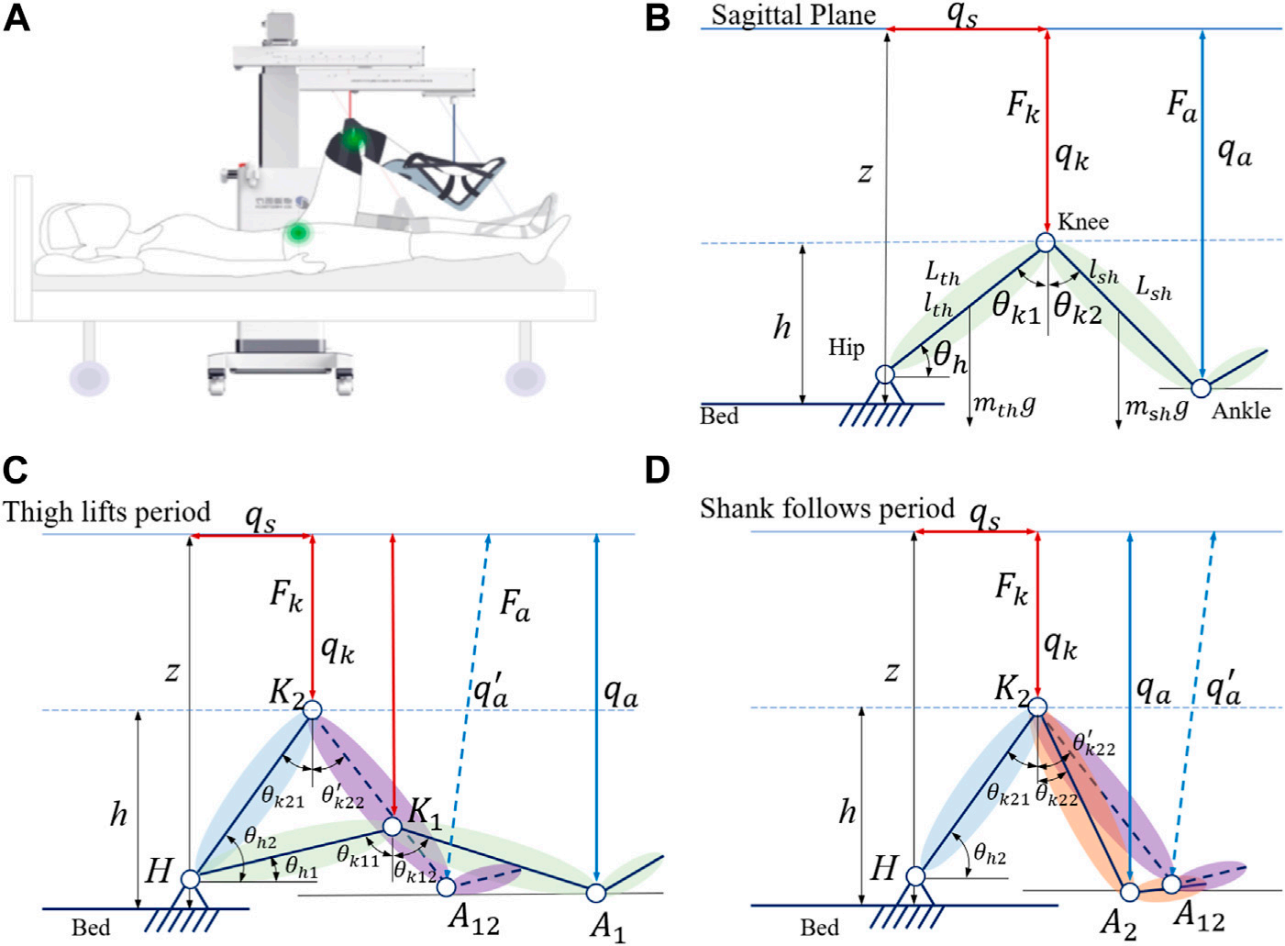
Modeling of the leg–machine coupling system in the sagittal plane. **(A)** General view of the human–machine coupling system. **(B)** Kinematic model of the leg–machine coupling system in the sagittal plane. *q*_*s*_ and *q*_*k*_ represent active actuation in the horizontal and vertical modules, respectively, which connect with the knee socket by a non-elastic cable. **(C)** Kinetic model of the leg–machine coupling system in the thigh lifting period. **(D)** Kinetic model of the leg–machine coupling system in the shank following period.

Synchronous flexion of the hip and knee in the sagittal plane (Figure 2A) is basic training for individuals with flaccidity after stroke. Thigh and shank lengths (*L_th_* and *L*_*sh*_) of the subjects can be set as predefined parameters in SmartSling. As shown in Figure 2B, at equilibrium, the human hip joint angle *θ*_*h*_ is

$$\theta_{h}=\sin^{-1}\frac{h}{L_{th}},$$ (1)

where the height of the knee joint *h* = *z* − *q*_*k*_, *h* is the height of the knee, *z* is the distance between the hip and vertical movement module, *q*_*k*_ is the sling movement of the vertical movement module, and *L_th_* is the length of the thigh. The knee joint angle *θ*_*k*_ is divided into two parts by the drive cable as

$$\theta_{k}=\theta_{k1}+\theta_{k2}$$ (2)

where *θ*_*k*1_ is the complementary angle of the hip joint angle, *θ*_*h*_, and *L*_*sh*_ is the length of the shank. Therefore, *θ*_*k*2_ can be calculated by the height of the knee joint as

$$\theta_{k2}=\sin^{-1}\frac{h}{L_{sh}}$$ (3)

Therefore, according to the active motion parameters (*q*_*s*_ and *q*_*k*_) of SmartSling, we can directly calculate the ideal kinematic state of the lower limb joints in real-time.

However, the cables can only be controlled under tension, and the inertia in the human body and the limb of the cable coupling system need to be taken into account. We found that the motion of the trained limb and machine is not completely synchronized when analyzing the position control. This phenomenon can be understood as follows: when starting, the cables are not fully tensioned and move, while the human body has not yet been stressed to move, and when braking, the cables are slack due to the inertia of human body movement. We need to consider the inertial motion of human extremities in the human–machine coupling system and establish dynamic equations to analyze the starting motion and braking motion process. The single-direction training cycle is divided into two states; in the first state, the machine actively slings the hip joint by lifting the thigh, while in the second state, the knee joint passively flexes, as shown in Figure 2D.

### 2.3 State 1: hip joint active slings

For the inpatients who cannot exert active hip joint torque, the thigh-related torque to the hip joint, *τ*_*hip*_, in the equilibrium state can be expressed as

$$\tau_{\mathrm{hip}}=J_{th}\alpha_{h}+F_{k}L_{th}\cos\theta_{h}+m_{th}gl_{th}\cos\theta_{h}$$ (4)

where *L_th_* is the length of the thigh, *l_th_* is the length from the hip joint to the center of mass of the thigh, *m_th_* is the mass of the thigh, *J_th_* is the moments of inertia of the thigh, and *F*_*k*_ is the tension force of the cable connected to the knee joint. The shank-related torque of the knee joint, *τ*_*knee*_, in the equilibrium state can be expressed as

$$\begin{array}{cc} \tau_{\mathrm{knee}} & =F_{a}\left(L_{th}\cos\theta_{h}+L_{sh}\sin\theta_{k2}\right)\\ & \;+m_{sh}g\left(L_{th}\cos\theta_{h}+l_{sh}\sin\theta_{k2}\right)+J_{sh}\alpha_{k} \end{array}$$ (5)

where *l*_*sh*_ is the length from the knee joint to the center of mass of the shank and foot and *L*_*sh*_ is the overall length of the shank and foot. Since the ankle socket binds to the shank and foot as a rigid connection, *m*_*sh*_ is the mass of the shank and foot, *J*_*sh*_ is the moment of inertia of the thigh and foot, and *F*_*a*_ is the tension force of the cable connected to the ankle joint.

From the equilibrium, the hip joint acceleration, *α*_*h*_, can be expressed as a function of the joint angle *θ*_*h*_,

$$\alpha_{h}=\frac{d^{2}\theta_{h}}{dt^{2}}=f\left(\theta_{h}\right)$$ (6)

By solving differential equations of *f* (*θ*_*h*_), we can get the relationship between the hip joint angle, *θ*_*h*_, and time, *t*, as

$$\theta_{h}\left(t\right)=F_{h}\left(t\right)$$ (7)

and the duration of hip rotation, Δ*t*_*h*_, can be written as

$$\Delta t_{h}=F_{h}^{-1}\left(\theta_{h2}\right)-F_{h}^{-1}\left(\theta_{h1}\right)$$ (8)

where $F_{h}^{-1}$ is the inverse function of *F*_*h*_(*t*) and *θ*_*h*1_ and *θ*_*h*2_ are the initial and terminal positions of the hip joint, respectively.

### 2.4 State 2: knee joint flex accompanies movement

In the second state, the knee joint flexes passively, which leads to its movement that is not as synchronous as that of the hip joint. The knee joint angle *θ*_*k*2_ has a hysteretic movement due to the fact that the cable connected to the ankle has a passive degree of freedom in a horizontal slide joint. The equilibrium condition at low speed during *θ*_*k*2_ movement can be written as

$$J_{sh}\alpha_{k}+F_{a}L_{sh}\cos\left(\theta_{k2}+\theta_{3}\right)+m_{sh}gl_{sh}\sin\theta_{k2}=0$$ (9)

where *θ*_3_ can be defined as

$$\theta_{3}=\cos^{-1}\left(\frac{q_{k}+L_{sh}\cos\theta_{k2}}{q_{a}}\right)$$ (10)

From the equilibrium, the knee joint acceleration, *α*_*k*_, can be expressed as a function of the joint angle *θ*_*k*2_,

$$\alpha_{k}=\frac{d^{2}\theta_{k2}}{dt^{2}}=r\left(\theta_{k2}\right)$$ (11)

By solving differential equations of *r* (*θ*_*k*2_), we can obtain the relationship between the second part of the knee joint angle, *θ*_*k*2_, and time, *t*, as

$$\theta_{k2}\left(t\right)=R_{k2}\left(t\right)$$ (12)

and the duration of the knee rotation, Δ*t*_*k*2_, can be written as

$$\Delta t_{k2}=R_{k2}^{-1}\left(\theta_{k22}^{\prime }\right)-R_{k2}^{-1}\left(\theta_{k22}\right)$$ (13)

where $R_{k2}^{-1}$ is the inverse function of *R*_*k*2_(*t*) and $\theta_{k22}^{\prime }$ and *θ*_*k*22_ are the initial and terminal positions of the second part of the knee joint in the shank following period, respectively.

The analysis of thigh lifting and shank following with position control of cables can illustrate the kinematic and kinetic aspects of the human–cable interaction during a single-sling process. The initialization hysteresis of the shank following the active sling procedure demonstrates that the human–cable interaction is unstable without force control.

### 2.5 Human–cable impedance controller

**FIGURE 3:**
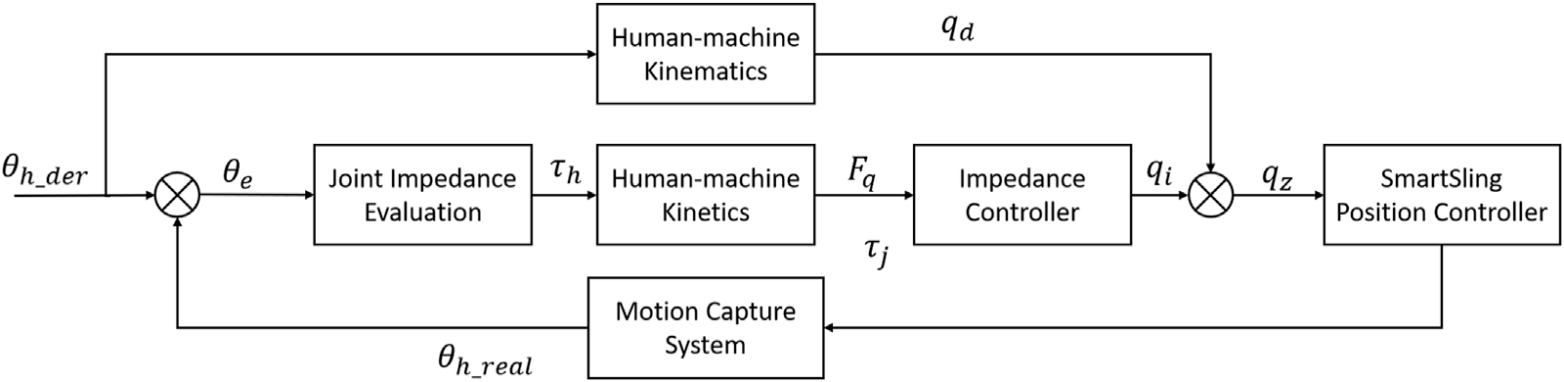
Impedance control diagram of the human–machine coupling system for SmartSling.

Here, we design a human–cable impedance controller (HCIC) to minimize the hysteresis of the shank following movement. The diagram of impedance control in the human–machine coupling system for SmartSling is shown in Figure 3. In the first state of active sling force, the kinetic equation can be established as

$$M\left(\theta \right)\ddot{\theta }+C\left(\theta ,\dot{\theta }\right)\dot{\theta }+G\left(\theta \right)=F_{k}+f_{d}$$ (14)

where *θ* is the vector containing hip and knee joint angles, *M*(*θ*) is the inertia matrix, $C\left(\theta ,\dot{\theta }\right)\dot{\theta }$ is the Coriolis matrix and centrifugal terms, *G*(*θ*) represents the gravity terms, *F*_*k*_ denotes the two cable tension forces, and *f*_*d*_ represents the lateral disturbances.

**FIGURE 4:**
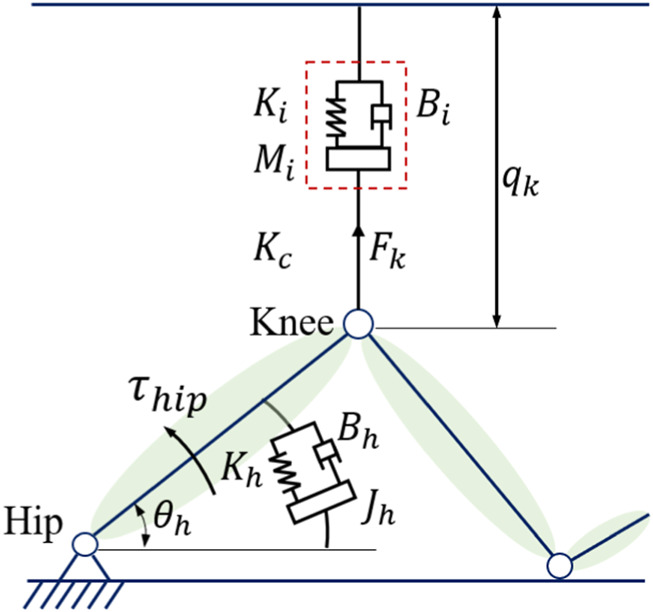
Bionic impedance model: the active sling force characterized by the human hip joint.

The active sling force is influenced by the human hip joint estimated impedance, as shown in Figure 4. During the impedance estimation process, the knee joint is kept in the state of maximum and the hip impedance in the sagittal plane can be identified from

$$\tau_{\mathrm{hip}}=J_{h}{\ddot{\theta }}_{h}+B_{h}{\dot{\theta }}_{h}+K_{h}\theta_{h}$$ (15)

where *J*_*h*_, *B*_*h*_, and *K*_*h*_ are the lower-limb intrinsic impedance parameters to be identified, while the joint angle *θ*_*h*_ and torque *τ*_*hip*_ are measurable or computable during the whole moving process.

With the identified lower-limb impedance parameters, the interaction force can be defined as

$$F_{k}=M_{i}{\ddot{q}}_{k}+B_{i}{\dot{q}}_{k}+K_{i}q_{k}$$ (16)

and the impedance parameters are defined as follows

$$\left[\begin{array}{c} M_{i}\\ B_{i}\\ K_{i} \end{array}\right]=\delta \left[\begin{array}{c} J_{h}\\ B_{h}\\ K_{h} \end{array}\right]/L_{th}cos\;\theta_{h}$$ (17)

where *δ* is the impedance gain and *q*_*k*_ can be traced back to *θ*_*h*_, as expressed in Eq. 1.

The hip joint error, *θ*_*e*_, can be defined as

$$\theta_{e}=\theta_{h\text{\_}der}-\theta_{h\text{\_}real}$$ (18)

where *θ*_*h*_*der*_ is the desired and *θ*_*h*_*real*_ is the real-time hip angle measured from a wearable motion capture system (MTI-1, Xsens, Netherlands). The error can be used to calculate the hip joint residential impedance parameters, as shown by Eq. 18. The impedance gain, *q*_*i*_, can be calculated with the impedance controller, as shown in Eq. 19. Finally, the output of the HCIC for the active cable length is the superposition of the desired length from human–machine kinematics, *q*_*d*_, and the impedance gain, *q*_*i*_, as

$$q_{k}=q_{d}+q_{i}$$ (19)

## 3 Experiments

The experiments had two objectives: first, observing the kinematics and kinetic performance of the subjects using SmartSling under the position control mode, and second, validating the impedance controller designed in this study to adjust and minimize the hysteresis of the shank following movement. Healthy patients were recruited and asked to be passive during the motion validation experiments to emulate bedridden patients with joint weakness. Overall, five healthy subjects (three males and two females, with mean age 26.6 years with a standard deviation (SD) of 2.07, height 1.72 m with SD 0.09, and weight 69.3 kg with SD 13.07) with no history of neurological impairments were tested. The segment value calculation referred to an anthropometric data distribution rule proposed by Kirtley (2004), as shown in Table 1. The subjects’ detailed information is given in Table 2. The Ethics Committee of the Changhai Hospital (Shanghai) approved this study. Written informed consent was obtained from all subjects.

**TABLE 1:** Anthropometric data.

Segment	Segment length, *l* (%BH)	Mass, *m* (%BM)	*l*_*CoM*_ (from the distal joint)	Radius of gyration *k*_*CoM*_
Thigh	25.4	9.9	56.7	0.3
Shank	23.3	4.6	57	0.3
Foot	11.7	1.4	50	0.48

BH, body height; BM, body mass; moment of Inertia, $I=m{\left(k_{\mathrm{CoM}}+l_{\mathrm{CoM}}\right)}^{2}$

**TABLE 2:** Subject information.

Subject number	Gender	Age (years)	BH (m)	BM (kg)	Thigh length (cm)	Shank length (cm)	Thigh mass (kg)	Shank mass (kg)	Thigh moment of inertia (*kgm* ^2^)	Shank moment of inertia (*kgm* ^2^)
1	M	27	1.783	73.9	45.2882	41.5439	7.3161	3.3994	253.2025	97.6329
2	F	25	1.611	58.4	40.9194	37.5363	5.7816	2.6864	163.3522	62.9827
3	M	25	1.855	90.2	47.117	43.2215	8.9298	4.1492	334.5148	128.9863
4	F	26	1.649	61.3	41.8846	38.4217	6.0687	2.8198	179.6481	69.2709
5	M	30	1.712	62.7	43.4848	39.8896	6.2073	2.8842	198.0596	76.3702

The experiments are divided into two main processes. 1) Under the position control mode, the robotic system is operated using three velocity levels for the vertical movement of the drive cable at the knee joint, corresponding to 0.05 m/s, 0.1 m/s, and 0.15 m/s, respectively. The time required for a complete set of hip and knee suspension training at different speeds was not synchronized with the position control mode. The average interval time was 5% training cycle time for the three training speeds. 2) In the HCIC mode, the active motion of SmartSling is adjusted in real-time according to the subject’s force because of the presence of human–robot interaction. The hip joint torque can be estimated by the HCIC as shown in Eq. 18, which can be a reference parameter for the functional movement evaluation. The speed of motion at a certain position is not determined for different subjects due to the differences in limb characteristics. We fixed only the lowest and highest positions of the drive cable. The obtained experimental results were mainly used to verify the synchronization enhancement of the HCIC for hip and knee joint linkage. The five subjects were asked to perform suspension training with and without active force, respectively, and each training session was carried out three times for each velocity level.

All experimental data exported from the SmartSling system were collected at a sampling rate of 100 Hz. The root mean square error (RMSE) of the experimental results was calculated for analysis. We calculated the active moments of the subjects in real-time through the human–machine coupled kinetic model. The kinetic data could not be temporarily compared and tested using third-party human motion analysis instruments. The normalized comparative analysis of the calculated active motion in humans was performed using SmartSling without making specific numerical comparisons due to the human moment calibration and reference data during sling training. The data of five stable sling training cycles are processed to verify the relationship between the calculation results of the locomotion identification system and the reference values. The RMSE *E*(*q*) is obtained as $E\left(q\right)=\sqrt{\frac{1}{n}\sum_{i=1}^{n}{\left(q-mean\left(q\right)\right)}^{2}}$ where *q* are the joint angles calculated by the locomotion identification system and *mean*(*q*) are the mean values of *q*. One-way analysis of variance (ANOVA) was conducted to compare the differences in the kinematic performance and estimated joint kinetics, with significance set at *p* ≤ 0.05.

## 4 Results

### 4.1 Kinematics under position control

**FIGURE 5:**
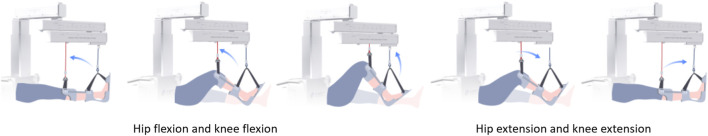
Training cycle schematics for SmartSling with the trained subject. The arrows indicate the directions of limb movement in the sagittal plane.

**FIGURE 6:**
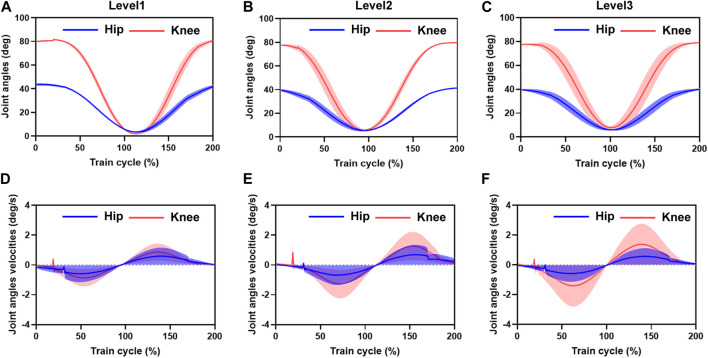
Mean and standard deviation of hip and knee motion for different training speed levels under position control without active force. **(A–C)** are the hip range of motion. The hip and knee motion data of the subjects revealed no significant differences in the joint range of motion. However, the RMSE of the hip and knee range of motion angles differed significantly with the increasing speed in motion. The distinct instability of the angular velocities can be observed with the increasing training velocity. **(D–F)** are the RMSE of the angular velocities of hip and knee motion varied with increasing training speed. Hip RMSE and the error bands increased significantly with increasing training speed. Knee RMSE and the error band increased with increasing training speed.

The entire training cycle was divided into joint flexion and extension movements, and each part was time-normalized into cycle percentage, as shown in Figure 5. The mean and standard deviation curves of hip and knee motion for different training levels under position control without active force are shown in Figures 6A–F. The hip range of motion was 5.3°–40.7°, 5.1°–42.9°, and 7.3°–40.0°, respectively, and no significant differences were found in the joint range of motion at different training speeds (*p* = 0.23). The knee range of motion was 4.3°–83.6°, 6.1°–81.2°, and 5.5°–82.5°, respectively, and no significant differences were found in the joint range of motion at different training speeds (*p* = 0.15). The hip and knee motion data of the subjects revealed no significant differences in the joint range of motion. However, the RMSE of hip and knee angles differed significantly with the increasing speed of training. The hip RMSE at the three training speeds was 2.7°, 3.5°, and 5.4°, respectively, significantly increasing by 29.63% (*p* = 0.029) and 44.35% (*p* = 0.013) with increasing training speed, respectively. The knee RMSE was 3.3°, 6.9°, and 12.4°, respectively, significantly increasing with increasing training speed by 52.17% (*p* = 0.015) and 44.35% (*p* = 0.008), respectively. The speed of motion in a defined human–machine motion chain is negligible for the joint range of motion, but the distinct instability of the angular velocities can be observed with the increase in training speed.

The angular velocities were obtained by the joint position differences. The sampling time interval was 0.01 s. The hip and knee joint angle results showed that the joint angle error bands caused by the increased training speed are amplified in the velocity calculation. A 5% training cycle time was reserved for the joint flexion and extension limits of each training cycle to mitigate the knee joint hysteresis problem before initiating the motion direction switch. The RMSE of the angular velocities of hip and knee motion varied significantly with increasing training speed, with a hip RMSE of 1.2 deg/s, 1.6 deg/s, and 1.7 deg/s for the three training speeds, respectively, and the error bands increasing significantly by 33.49% (*p* = 0.013) and 6.25% (*p* = 0.028) with increasing training speed, respectively. The knee RMSE was 1.3 deg/s, 1.8 deg/s, and 2.1 deg/s, respectively, and the error band significantly expanded with increasing training speed by 38.46% (*p* = 0.021) and 14.28% (*p* = 0.017), respectively. At the beginning of the draft training, we noticed some oscillations in the joint motion speed, with maximum oscillation of 0.72 deg/s, 1.13 deg/s, and 0.37 deg/s in the hip joint and 0.21 deg/s, 0.26 deg/s, and 0.41 deg/s in the knee joint, respectively. The source of these oscillations was mainly due to the unstable motion caused by the human-driven cable interaction force, which was not considered in the human–machine coupling system.

**FIGURE 7:**
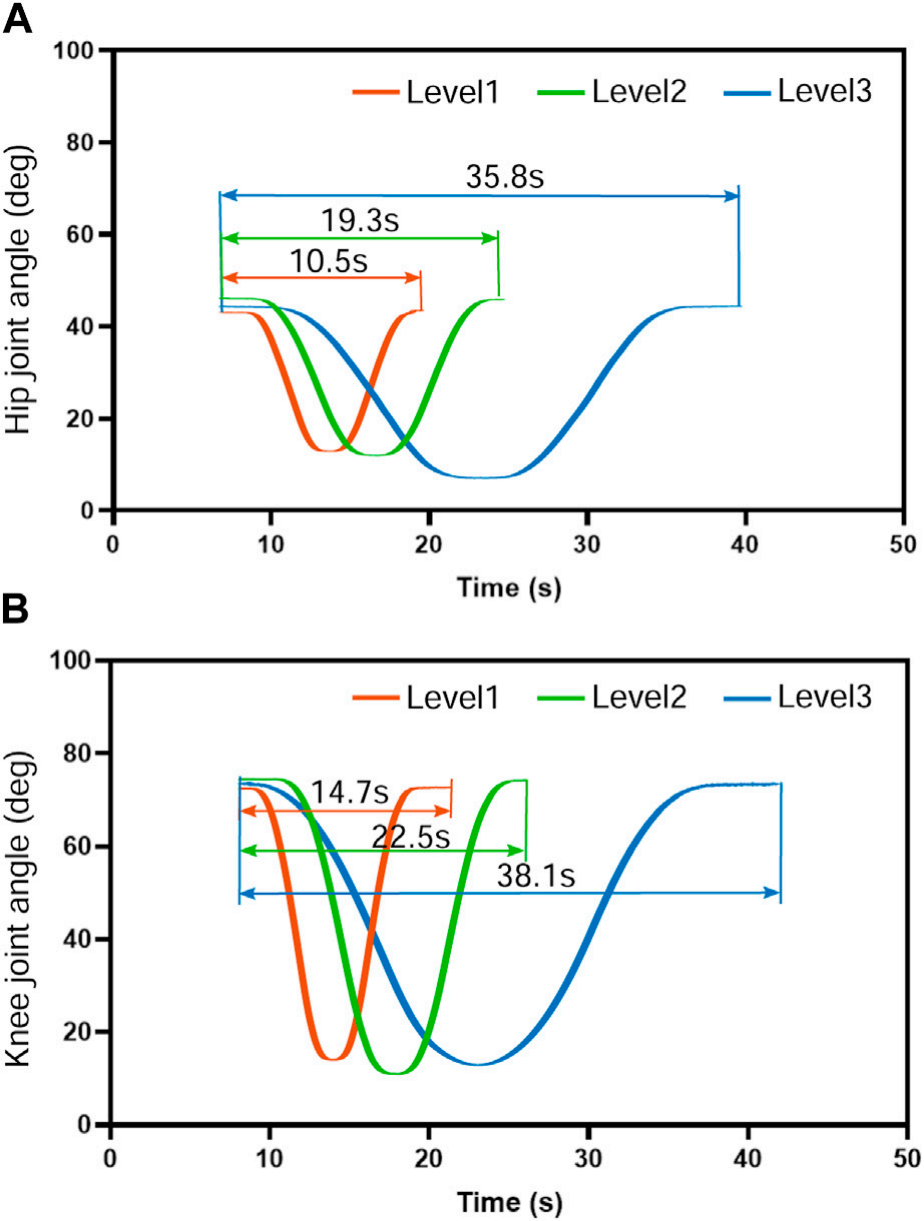
Movement duration of the five subjects’ hip and knee joints over 10 training cycles under the position control mode for the three training speed levels. The knee joint moved at an average speed of 4.2 s, 3.2 s, and 2.6 s, respectively, slower than the hip joint. The knee hysteresis decreased with increasing training speed with no significant association (*p* >0.1) between the training level and knee hysteresis. As the training speed level increased, the hip and knee movement time remained consistent. The hip movement time decreased by 8.8 s and 16.5 s, respectively, and the knee movement time decreased by 7.8 s and 15.6 s, respectively.

Figure 7 demonstrates the mean movement duration of the five subjects’ hip and knee joints over 10 training cycles under the position control mode for the three training speed levels. The knee joint lagged with an average of 4.2 s, 3.2 s, and 2.6 s, respectively, after the hip joint completed the movement, which means that the hip and knee joints steadily experienced asynchrony problems during the suspension training. This knee hysteresis decreased with increasing training speed, but we did not find a significant association between the training level and knee hysteresis in the one-way ANOVA. However, as the training speed level increased, the hip movement time decreased by 8.8 s and 16.5 s, respectively (Figure 7A), and the knee movement time decreased by 7.8 s and 15.6 s, respectively (Figure 7B), while the hip and knee movement time remained consistent. This trend was consistent with the change time of the human–machine coupled hip motion by calculating the vertical motion time of the active cable at different speed levels. Therefore, the motion trajectory control and time planning under the position control mode are stable.

### 4.2 Human hip joint impedance estimation

**FIGURE 8:**
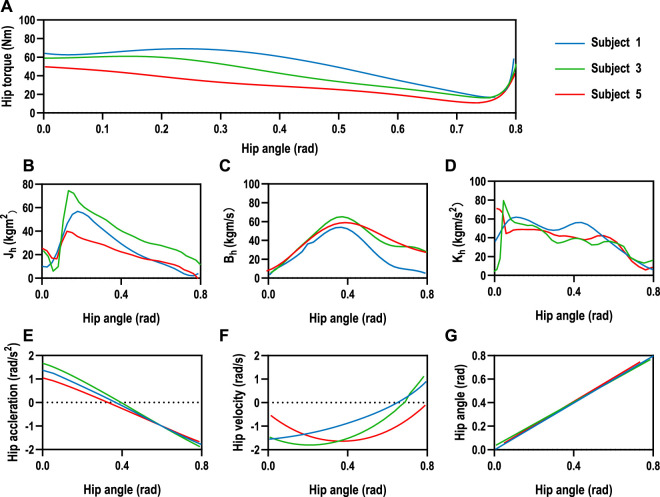
Human hip joint impedance estimation. **(A)** The mean joint torques of the three subjects were 22.35*Nm*, 31.99*Nm*, and 27.64*Nm*, respectively, which were positively correlated with their body weight. The range of motion of the hip joint was controlled from 0 to 45° during the estimation progress to facilitate experimental data comparison between different subjects. **(E–G)** The human kinematic performances of the three subjects are relatively stable. However, intrinsic differences existed in body weight and lower-limb proportions among the three subjects. **(B–D)** The lower-limb rotational inertia showed significant differences due to the consequential differences in weight and lower limb dimensions among the three subjects, while the trend and range of hip damping and stiffness coefficients were similar for the three subjects, with an average joint damping of 37±2.3 *kgm*/*s*, and the average stiffness was 40±1.6 *kgm*/*s* ^2^.

The hip joint impedance coefficients of the subjects are estimated for the human–machine coupling system controller with Eq. 14. The mean values of the relevant parameters were estimated in real-time for 10 sets of hip tests on three male subjects, as shown in Figure 8. The subjects were asked not to exert moment on the hip joint during the test but to rely only on the driven force of SmartSling to measure the passive impedance coefficients of the hip joints. The mean joint torques of the three subjects were 22.35*Nm*, 31.99*Nm*, and 27.64*Nm*, respectively, which were positively correlated with their body weight (Figure 8A). The range of motion of the hip joint was controlled from 0 to 45° during the estimation progress to facilitate experimental data comparison between different subjects. Thus, the kinematic performances of the three subjects are relatively stable, as shown in Figures 8E–G. However, due to the intrinsic differences in body weight and lower-limb proportions among the three subjects, the lower-limb rotational inertia shown in Figures 8B–D exhibited consequential differences. The lower-limb rotational inertia of subject 3 was 0.21*Nms* ^2^/*kg* larger than that of subject 1 when the body weight is the reference index. The trend and range of hip damping and stiffness coefficients were similar for the three subjects, with an average joint damping of 37 ± 2.3 *kgm*/*s*, and the average stiffness was 40 ± 1.6 *kgm*/*s* ^2^.

### 4.3 Knee and ankle trajectory optimization

**FIGURE 9:**
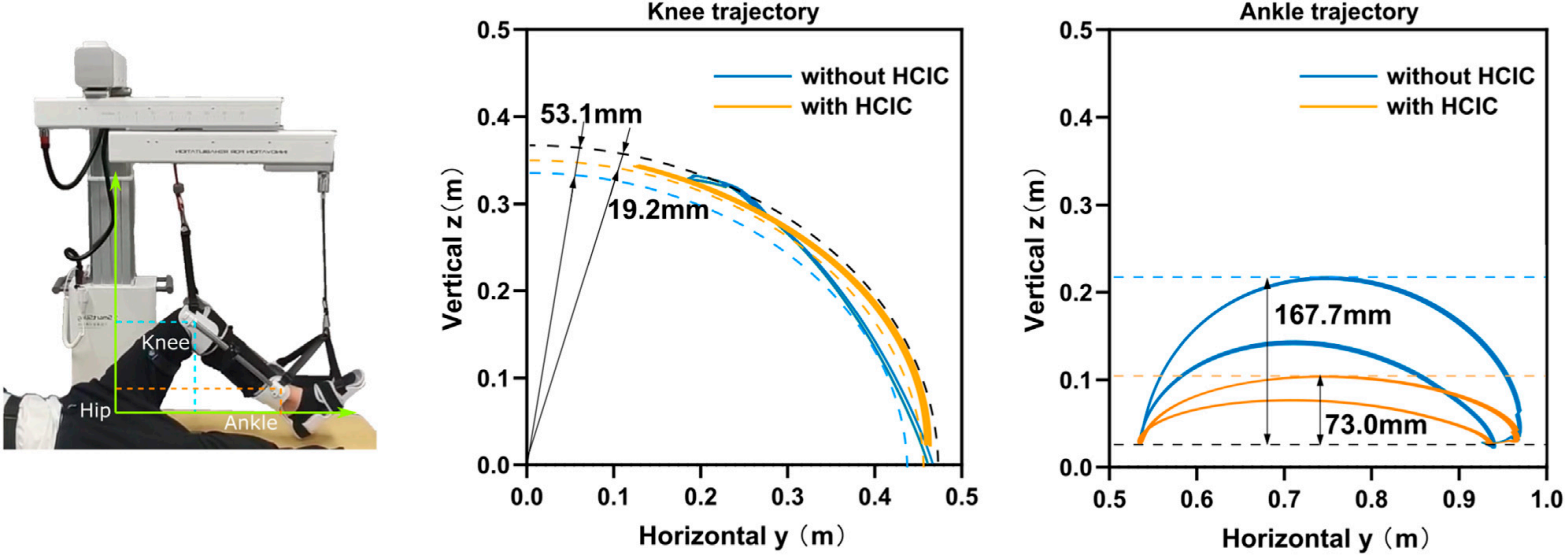
The motion trajectories of the knee and ankle joints within the Cartesian space can be utilized for movement assessment. The knee and ankle joint trajectories of subject 1 in the sagittal plane under position control and HCIC training modes are denoted by blue and orange curves. The knee motion has radial oscillation in addition to the circular arc motion around the hip joint due to the instability of the human–machine coupled motion. We use the two circles shown by dashed lines to envelop the knee trajectory under two training modes. The width of the knee oscillation annulus under position control is 53.1 *mm*, and the width of the oscillation annulus under HCIC control is 19.2 *mm*. The whole trajectory profile is closer to a circular arc with a decrease of 63.84% in the knee radial oscillation. The ankle range of motion in the vertical direction was 167.7 *mm* under position control and 73.0 *mm* under HCIC control, and the ankle jump range was reduced by 56.47% under the same horizontal motion start point condition.

The motion trajectories of the knee and ankle joints within the Cartesian space can be utilized for movement assessment in addition to the aforementioned kinematic and dynamic parameters of the lower limb joints. The hip joint was used as the origin in the sagittal plane, with the horizontal direction as the horizontal coordinate and vertical direction as the vertical coordinate, as shown in Figure 9. We set the impedance gain *δ* = 0.8 for the HCIC of subject 1. The knee and ankle joint trajectories of subject 1 in the sagittal plane under position control and HCIC training modes are denoted by blue and orange curves, respectively. The knee joint is theoretically an arc of a circle. Still, the knee motion has radial oscillation in addition to the circular arc motion around the hip joint due to the instability of the human–machine coupled motion. We use the two circles shown by dashed lines to envelop the knee trajectory under two training modes. The width of the knee oscillation annulus under position control is 53.1 *mm*, and the width of the oscillation annulus under HCIC control is 19.2 *mm*. The whole trajectory profile is closer to a circular arc with a decrease of 63.84% in the knee radial oscillation. The ankle joint cannot continuously extend the horizontal movement since the ankle joint-connected cable of SmartSling is a follower joint and moves only in the horizontal direction. As the follower joint of the knee joint, the oscillation effect of the knee joint is amplified at the ankle joint. The horizontal start and end positions are determined and are, therefore, not affected by the control strategy. However, the ankle joint trajectory during the motion shows different effects in the vertical direction of cable traction due to the limb oscillation in the continuous motion because of the different sling control strategies. The ankle range of motion was 167.7 *mm* under position control and 73.0 *mm* under HCIC control, and the ankle jump range was reduced by 56.47% under the same horizontal motion start point condition. The ankle and knee joint trajectory results demonstrated that the lower-limb rehabilitation training of bedridden individuals could be performed using SmartSling stably and smoothly.

### 4.4 Kinematics optimization with HCIC

**FIGURE 10:**
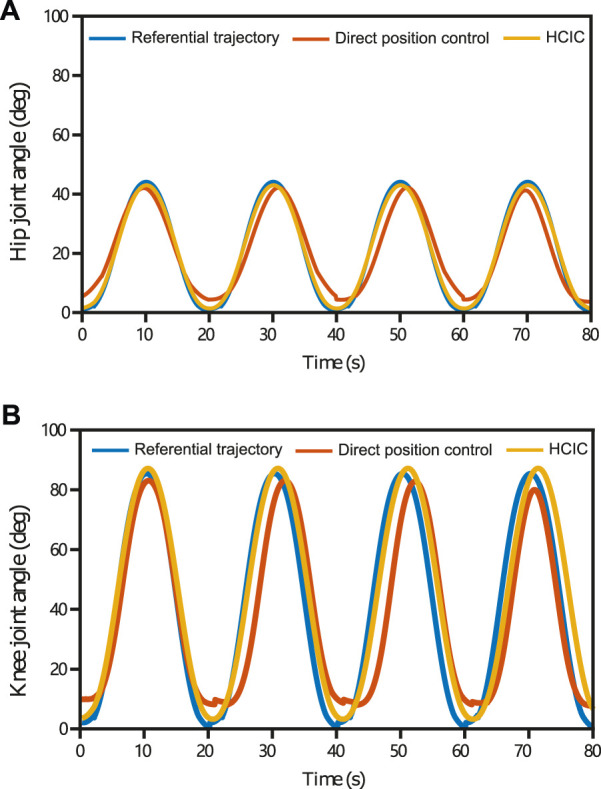
Hip and knee joints’ kinematic performance in four training cycles (subject 1) under position control and HCIC. The target range of motion of the hip joint was from 0 to 45.0°, and the corresponding motion calculation could yield a knee range of motion from 0 to 84.3°. The ultimate range of motion of the hip and knee joints under position control was close to 95.3% and 92.7% of the reference trajectories, respectively. Under position control, the hip extension was incomplete (the average ultimate extension was 3.5°±0.7°), and the knee extension was incomplete (the average ultimate extension was 10.9°±1.3°). The HCIC effectively restored the hip and knee range of motion to 98.4% and 96.5% of the reference range, respectively. The HCIC reduced knee hysteresis by an average of 2.1 s per training cycle compared to position control.

One of the objectives of the experiments is to observe the kinematic performance of the subjects to compare the effectiveness of SmartSling under the position control mode and HCIC. Healthy patients with no active joint movement can perform the same function at this experimental stage as bedridden patients with joint weakness. The kinematic performance of subject 1 (Figure 10) was analyzed to demonstrate the superiority of the HCIC compared to direct positional control. The hip and knee joints of the five subjects were analogous. The target range of motion of the hip joint was from 0 to 45.0°, and the corresponding calculation of motion could yield a knee range of motion from 0 to 84.3°. In addition, the theoretical motion states of the two joints were synchronized during the sling training. The ultimate range of motion of the hip and knee joints under position control was close to 95.3% and 92.7% of the reference trajectories, respectively. However, the hip extension was incomplete (the average ultimate extension was 3.5° ± 0.7°), and the knee extension was incomplete (the average ultimate extension was 10.9° ± 1.3°). The HCIC effectively restored the hip and knee range of motion to 98.4% and 96.5% of the reference range, respectively. In addition, in terms of correcting hip and knee synchronization, as shown in Figure 10B, the HCIC reduced motor knee hysteresis by an average of 2.1 s per training cycle compared to position control, which accounts for 10.3% of a single training cycle.

## 5 Discussion

We designed, built, and tested a cable-driven bedside lower-limb rehabilitation robot to assist patients in patient wards who cannot be transferred over a wide range but are in urgent need of lower-extremity rehabilitation training. The kinematics of the proposed bedside rehabilitation robot under position control was verified with healthy subjects. The lower-limb multi-joint motion has the problem of motion asynchrony due to the limitation of the cable drive itself. Although cable driving is a flexible human–machine interaction method, the human–machine coupled system tended to be unstable under position control in the actual operation process without considering the human joint impedance. Therefore, we added force sensors at one end of the driven cables and proposed a human–machine coupling kinetic model to dynamically identify the impedance coefficients of human lower limbs in the joint space. We designed a human–cable impedance controller based on the identified human joint impedance coefficients. Subject-training experimental tests demonstrated that the proposed human–computer coupled impedance controller improved the synchronization of the lower limbs over the joints and the stability of the human–cable coupled system and restored the range of motion of lower limb joints.

Compared with previous cable-driven rehabilitation robots, the force interaction of the human–cable coupled system proposed in this study can improve the system stability. Cable-driven rehabilitation is inherently flexible as a particular human–machine interaction. Still, the application of force-open loop makes it challenging to avoid inertial movement because of the mismatch between human and robot system impedance characteristics. SET is an unstable, open-chain, and closed-chain movement performed using a suspension aid to improve the stability of the body’s core muscles (Oh and Kwon, 2017). The stability of human motion with SmartSling was analyzed in two aspects: end-joint trajectory and joint angle. Analysis of the knee and ankle joint trajectories of the tested subject in the sagittal plane under position control and HCIC training modes showed that the knee motion has radial oscillation in addition to the circular arc motion around the hip joint due to the instability of the human–machine coupled motion. The trajectory profile is closer to a circular arc with a decrease of 63.84% of the knee radial oscillation. The ankle joint cannot continuously extend the horizontal movement since the ankle joint-connected cable of SmartSling is a follower joint and moves only in the horizontal direction. The oscillation effect of the knee joint is amplified at the ankle joint as the knee joint follows the hip joint. The horizontal start and end positions are determined and are, therefore, not affected by the control strategy. However, the ankle joint trajectory during the motion shows different effects in the vertical direction of cable traction due to the limb oscillation in the continuous motion because of the different sling control strategies.

For a bedside rehabilitation robot, rigid driving is not safe enough for a patient in the bedside stage. The existing direct drive of distal joints, such as the ankle joint through the end of the robot arm, is dangerous for the patient without real-time feedback on the torque and angle of the intermediate joints for rehabilitation training (Tsuda, 2006; KUKA, 2008). In contrast, our proposed robot’s coordinated drive for each joint is based on flexible force interaction. The closed-loop human–machine dynamic model calculates the human joint torque in real-time. The experimental results show that this rehabilitation training strategy is safe and effective.

The coupled modeling method of the robotic system proposed in this study provides technical references for other cable-driven robotic designs in human–machine interaction force perception, human static characteristic measurement, and joint motion capability assessment. Currently, SmartSling has limitations for multi-posture lower-extremity training (lying and lateral postures) and standing balance training, according to SET. SmartSling mainly targets lower-extremity limb movement training in the flaccid paralysis phase but is considered from the perspective of flexible human–machine interaction features and easily replaceable wearable accessories. It is expandable to upper-extremity training and even to whole-body training through multi-robot formation, which shows great potential for rehabilitation training in narrow patient wards. In addition, the stability of the human–machine coupling system through the dynamic identification of human–machine interaction force is a potential theoretical research and application scenario in the direction of cable-driven robots, especially for neural rehabilitation robots.

## 6 Conclusion

This cable-driven lower-limb rehabilitation robot is designed with closed-chain kinematics and kinetics for the human–machine coupled system. It can provide professional and efficient lower-limb rehabilitation training at the bedside for stroke patients. The recognized human joint active parameters from the kinematic and kinetic models will be the clinical references for the next stage of rehabilitation training. We are currently planning on using the rehabilitation robot to investigate the clinical research with cooperated rehabilitation departments, especially for bedside SET training research. In the next research stage, the proposed impedance matching algorithm will be extended to upper-limb rehabilitation training. The flexible human–machine interaction method in this paper is being investigated for application to training scenarios in physical therapy and occupational therapy, more than just the bedside.

## Data Availability

The original contributions presented in the study are included in the article/Supplementary Material; further inquiries can be directed to the corresponding authors.
